# Supplementary material for: Pyruvate Dehydrogenase Kinase 1 inhibition mediated oxidative phosphorylation enhancement in cartilage promotes osteoarthritis progression
Source: BMC Musculoskelet Disord. 2023 Jul 20;24:597. doi: 10.1186/s12891-023-06585-6 (PMC10357736; doi:10.1186/s12891-023-06585-6)
Supplement: Supplementary file 1 — Additional file 1. [file 12891_2023_6585_MOESM1_ESM.zip › MF.Gsea.1653627316242/GOMF_COA_HYDROLASE_ACTIVITY.html]

Details for gene set GOMF\_COA\_HYDROLASE\_ACTIVITY[GSEA]

|  || Dataset | 55457\_collapsed\_to\_symbols.cls.cls #OA\_versus\_normal.cls.cls #OA\_versus\_normal\_repos |
| Phenotype | cls.cls#OA\_versus\_normal\_repos |
| Upregulated in class | OA |
| GeneSet | GOMF\_COA\_HYDROLASE\_ACTIVITY |
| Enrichment Score (ES) | 0.46604648 |
| Normalized Enrichment Score (NES) | 1.412399 |
| Nominal p-value | 0.08352145 |
| FDR q-value | 0.31261966 |
| FWER p-Value | 1.0 |
Table: GSEA Results Summary

  

Fig 1: Enrichment plot: GOMF\_COA\_HYDROLASE\_ACTIVITY      
 Profile of the Running ES Score & Positions of GeneSet Members on the Rank Ordered List

  

| SYMBOL | TITLE | RANK IN GENE LIST | RANK METRIC SCORE | RUNNING ES | CORE ENRICHMENT || 1 | ACOT13 | acyl-CoA thioesterase 13 [Source:HGNC Symbol;Acc:HGNC:20999] | 51 | 0.989 | 0.1810 | Yes |
| 2 | ACOT7 | acyl-CoA thioesterase 7 [Source:HGNC Symbol;Acc:HGNC:24157] | 971 | 0.524 | 0.2077 | Yes |
| 3 | PPT1 | palmitoyl-protein thioesterase 1 [Source:HGNC Symbol;Acc:HGNC:9325] | 1042 | 0.508 | 0.2974 | Yes |
| 4 | ACOT8 | acyl-CoA thioesterase 8 [Source:HGNC Symbol;Acc:HGNC:15919] | 1088 | 0.500 | 0.3873 | Yes |
| 5 | HIBCH | 3-hydroxyisobutyryl-CoA hydrolase [Source:HGNC Symbol;Acc:HGNC:4908] | 1526 | 0.424 | 0.4328 | Yes |
| 6 | OLAH | oleoyl-ACP hydrolase [Source:HGNC Symbol;Acc:HGNC:25625] | 2673 | 0.283 | 0.3967 | Yes |
| 7 | ACAA2 | acetyl-CoA acyltransferase 2 [Source:HGNC Symbol;Acc:HGNC:83] | 2779 | 0.272 | 0.4395 | Yes |
| 8 | ABCD2 | ATP binding cassette subfamily D member 2 [Source:HGNC Symbol;Acc:HGNC:66] | 3031 | 0.246 | 0.4660 | Yes |
| 9 | ACOT2 | acyl-CoA thioesterase 2 [Source:HGNC Symbol;Acc:HGNC:18431] | 4046 | 0.152 | 0.4157 | No |
| 10 | ACOT9 | acyl-CoA thioesterase 9 [Source:HGNC Symbol;Acc:HGNC:17152] | 5451 | 0.037 | 0.3137 | No |
| 11 | ACSBG2 | acyl-CoA synthetase bubblegum family member 2 [Source:HGNC Symbol;Acc:HGNC:24174] | 5970 | -0.002 | 0.2739 | No |
| 12 | ACOT11 | acyl-CoA thioesterase 11 [Source:HGNC Symbol;Acc:HGNC:18156] | 7746 | -0.137 | 0.1619 | No |
| 13 | ABCD3 | ATP binding cassette subfamily D member 3 [Source:HGNC Symbol;Acc:HGNC:67] | 8877 | -0.223 | 0.1158 | No |
| 14 | ABCD1 | ATP binding cassette subfamily D member 1 [Source:HGNC Symbol;Acc:HGNC:61] | 8930 | -0.226 | 0.1541 | No |
| 15 | BAAT | bile acid-CoA:amino acid N-acyltransferase [Source:HGNC Symbol;Acc:HGNC:932] | 10523 | -0.376 | 0.1009 | No |
| 16 | PLA2G6 | phospholipase A2 group VI [Source:HGNC Symbol;Acc:HGNC:9039] | 11128 | -0.446 | 0.1375 | No |
Table: GSEA details [plain text format]

  

Fig 2: GOMF\_COA\_HYDROLASE\_ACTIVITY      
 Blue-Pink O' Gram in the Space of the Analyzed GeneSet

  

Fig 3: GOMF\_COA\_HYDROLASE\_ACTIVITY: Random ES distribution      
 Gene set null distribution of ES for **GOMF\_COA\_HYDROLASE\_ACTIVITY**
